# Supplementary material for: Feature-specific reaction times reveal a semanticisation of memories over time and with repeated remembering
Source: Nat Commun. 2021 May 26;12:3177. doi: 10.1038/s41467-021-23288-5 (PMC8155072; doi:10.1038/s41467-021-23288-5)
Supplement: Supplementary file 1 — Supplementary Information [file 41467_2021_23288_MOESM1_ESM.pdf]

Supplementary Information

Feature-specific reaction times reveal a semanticisation of memories over time and with repeated remembering

Julia Lifanov[1], Juan Linde-Domingo[2], & Maria Wimber[1, 3]

1) School of Psychology and Centre for Human Brain Health (CHBH), University of Birmingham, Birmingham, United Kingdom.
2) Center for Adaptive Rationality, Max Planck Institute for Human Development, Berlin, Germany.
3) Institute of Neuroscience & Psychology and Centre for Cognitive Neuroimaging (CCNi), University of Glasgow, Glasgow, United Kingdom.

Supplementary Methods

Subsampling method to compare the RT gaps between retrieval and restudy on day 2

To assure that the differential RT gap between groups is not a consequence of the unequal sample sizes, we randomly drew 5000 subsamples of size n = 24 of the repeated retrieval group, to equalize the group size to the one of the restudy group (n = 24). We then computed the z-score between the mean RT gap in the restudy group and the distribution of mean RT gaps of the resampled retrieval groups. First, we confirmed that, not surprisingly, the mean RT gaps in the subsampled retrieval groups (n = 24) distributed around the mean RT gap observed for the larger sample (n = 49). Across the 5000 sub-samples, the perceptual-conceptual RT gap in the retrieval group had a mean of 290 msec (95% *CI* = [203 msec to 376 msec]). Critically, the mean RT gap in the restudy group (*mean* = 83 msec) showed no overlap with this confidence interval, and in fact we found zero cases amongst the 5000 subsamples where the restudy RT gap was equal to, or larger than, the retrieval RT gap. Comparing the restudy gap to the empirical distribution of retrieval RT gaps thus resulted in a highly significant z-score (z = -3.95, p < .01) and an empirical p-value <.01. This result confirms that the stronger semanticization in the repeated retrieval than restudy group also holds with equal sample sizes (Supplementary Fig. 1).

Subsampling method in testing effect analysis

We also wanted to make sure that the significant accuracy difference between groups, on the written paper and pencil test, is not influenced by the difference in sample sizes. We randomly drew 5000 subsamples from the repeated retrieval group with size *n* = 24, to equate the restudy group, and then computed the z-score between the restudy group mean and the retrieval sample distribution, both for specific and coarse responses. We found that the restudy accuracy significantly differed from the repeated retrieval accuracy distribution for both coarse (*z-score* = -3.63, *p* < .01, empirical *p* < .01) and specific accuracies (*z-score* = -3.45, *p* < .01, empirical *p* < .01). For both scoring methods, the mean accuracies of the restudy group (M_{coarse}= 0.20; $M_{specific}$= 0.16) were outside the 95% confidence intervals (CI_{coarse}= [0.25 to 0.34]; $CI_{specific}$= [0.21 to 0.29]), and in fact entirely outside the re-sampled retrieval distributions. This finding confirms that a robust testing effect is present in our data also when equating sample sizes, based on the commonly used cued recall accuracies (Supplementary Fig. 1).

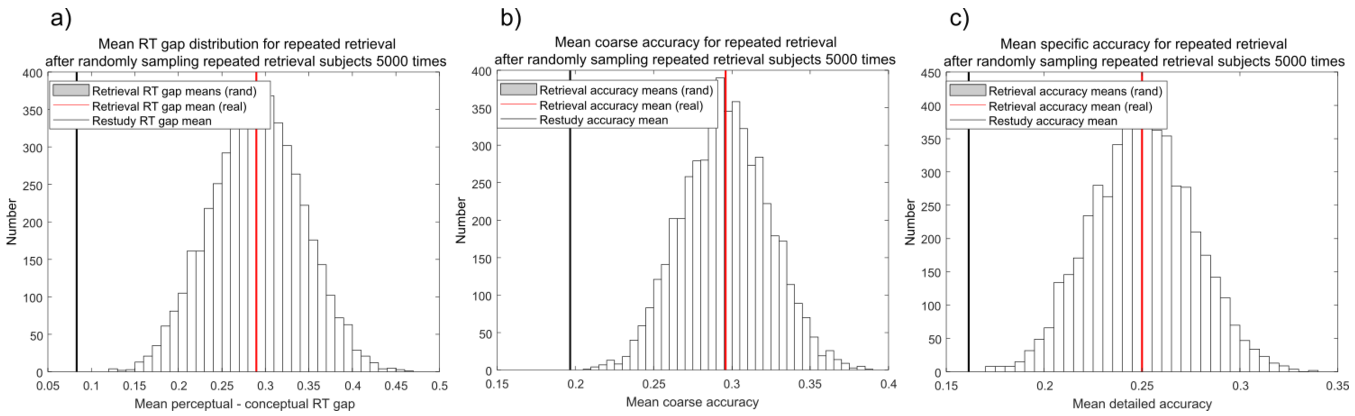

Supplementary Figure 1. Subsampling methods showing higher memory performance and perceptual-conceptual reaction time (RT) gap in retrieval on day 2. A) The distribution of mean RT gaps after drawing subsamples of the repeated retrieval group 5000 times shows that repeated retrieval yields a stronger semanticisation than restudy in 100% of the cases, even when using equally sized samples (one-sided *empirical p* = .00; *z* = -3.95, *p* = .00). B-C) The distribution of mean sheet response accuracies after drawing subsamples of the repeated retrieval group 5000 times shows that repeated retrieval leads to better accuracies than restudy according to (b) coarse (one-sided *empirical p* =.00; *z* = -3.63, *p* = .00) and (c) specific scoring (one-sided *empirical p* =.00; *z-score* = -3.45, *p* = .00). The black line represents restudy data, the red line retrieval data, and the distribution is created by randomly drawing subsamples from the retrieval data with *N* = 49 independent subjects in the retrieval group and *n* = 24 independent subjects in the restudy group. Source data are provided as a Source Data file.

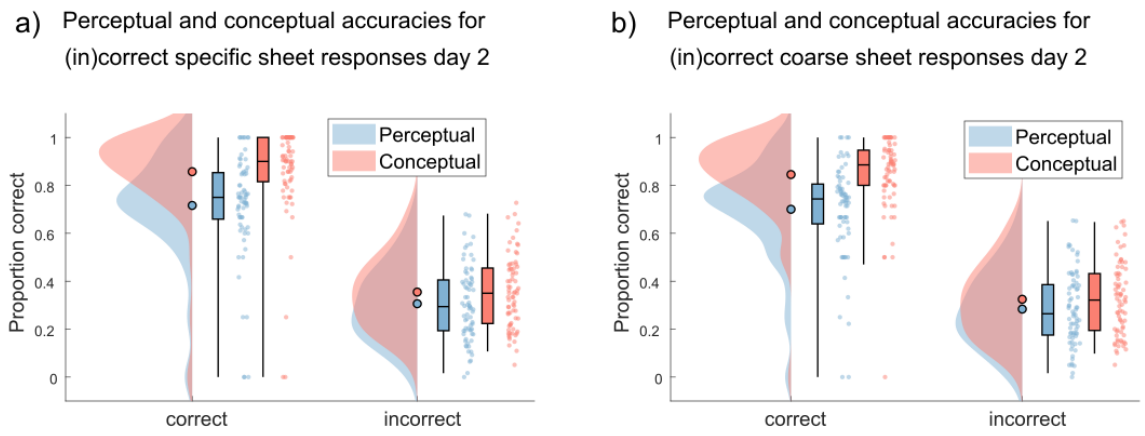

Supplementary Figure 2. Perceptual and conceptual accuracy performance for (in)correct specific and coarse written cued recall responses on day 2. Associations that are remembered in the cued recall test yield a stronger perceptual-conceptual accuracy gap in the task (two-sided $t(71) = 3.08$, $p = .00$, $CI = [.03, .15]$ with the specific scoring approach, two-sided $t(71) = 3.95$, $p = .00$, $CI = [.05, .16]$ with the coarse scoring approach). Task accuracies from cycle 3 in the repeated retrieval group have been categorized into correct/incorrect specific (a) and coarse (b) response sheets. Filled circles represent the overall mean, boxplots represent median and 25th and 75th percentiles; whiskers represent 2nd and 98th percentile; dots represent the means of individual subjects. Blue represents perceptual, red conceptual responses. N = 73 independent subjects. Source data are provided as a Source Data file.
