# Supplementary material for: Feature-specific reaction times reveal a semanticisation of memories over time and with repeated remembering
Source: Nat Commun. 2021 May 26;12:3177. doi: 10.1038/s41467-021-23288-5 (PMC8155072; doi:10.1038/s41467-021-23288-5)
Supplement: Supplementary file 3 — Reporting Summary [file 41467_2021_23288_MOESM3_ESM.pdf]

nature research

Corresponding author(s): Julia Lifanov

Last updated by author(s): Mar 25, 2021

Reporting Summary

Nature Research wishes to improve the reproducibility of the work that we publish. This form provides structure for consistency and transparency in reporting. For further information on Nature Research policies, see our Editorial Policies and the Editorial Policy Checklist.

Statistics

For all statistical analyses, confirm that the following items are present in the figure legend, table legend, main text, or Methods section.

n/a	Confirmed	
☐	☒	The exact sample size (*n*) for each experimental group/condition, given as a discrete number and unit of measurement
☐	☒	A statement on whether measurements were taken from distinct samples or whether the same sample was measured repeatedly
☐	☒	The statistical test(s) used AND whether they are one- or two-sided<br>*Only common tests should be described solely by name; describe more complex techniques in the Methods section.*
☒	☐	A description of all covariates tested
☐	☒	A description of any assumptions or corrections, such as tests of normality and adjustment for multiple comparisons
☐	☒	A full description of the statistical parameters including central tendency (e.g. means) or other basic estimates (e.g. regression coefficient) AND variation (e.g. standard deviation) or associated estimates of uncertainty (e.g. confidence intervals)
☐	☒	For null hypothesis testing, the test statistic (e.g. *F*, *t*, *r*) with confidence intervals, effect sizes, degrees of freedom and *P* value noted<br>*Give P values as exact values whenever suitable.*
☒	☐	For Bayesian analysis, information on the choice of priors and Markov chain Monte Carlo settings
☒	☐	For hierarchical and complex designs, identification of the appropriate level for tests and full reporting of outcomes
☒	☐	Estimates of effect sizes (e.g. Cohen's *d*, Pearson's *r*), indicating how they were calculated

Our web collection on statistics for biologists contains articles on many of the points above.

Software and code

Policy information about availability of computer code

Data collection	For the data collection we used: Matlab 2017a (www.mathworks.com), the Psychophysics Toolbox Version 3 (Brainard, 1997; Pelli, 1997; Kleiner, Brainard, Pelli, Ingling, Murray & Broussard, 2007), and a customized paradigm code provided by Linde-Domingo, Treder, Kerrén, & Wimber (2019).
Data analysis	For the data analysis we used: Matlab 2017a (www.mathworks.com); a customized Mathworks code by Caplette (2020); a customized Mathworks code by Schurger (2020); raincloudplot Version 1.1 (https://github.com/RainCloudPlots/); the G*power 3.1 (Faul, Erdfelder, Buchner, & Lang, 2009); ColorBrewer 2.0 (from www.ColorBrewer.org) and ColorBrewer schemes 2.0 for Matlab (Charles, 2020) and Inkscape 1.0.1 (https://inkscape.org/). The code that supports the findings of this study is available under https://doi.org/10.17605/OSF.IO/WP4FU.

For manuscripts utilizing custom algorithms or software that are central to the research but not yet described in published literature, software must be made available to editors and reviewers. We strongly encourage code deposition in a community repository (e.g. GitHub). See the Nature Research guidelines for submitting code & software for further information.

Data

Policy information about availability of data

All manuscripts must include a data availability statement. This statement should provide the following information, where applicable:

- Accession codes, unique identifiers, or web links for publicly available datasets
- A list of figures that have associated raw data
- A description of any restrictions on data availability

Source data are provided with this paper. The retrieval and restudy data files that support the findings of this study is available in “Retrieval_group” and “Restudy_group” respectively with the identifier https://doi.org/10.17605/OSF.IO/WP4FU. Stimulus material can be found in the BOSS database (https://sites.google.com/site/bosstimuli/home).

Field-specific reporting

Please select the one below that is the best fit for your research. If you are not sure, read the appropriate sections before making your selection.

☐ Life sciences ☒ Behavioural & social sciences ☐ Ecological, evolutionary & environmental sciences

For a reference copy of the document with all sections, see nature.com/documents/nr-reporting-summary-flat.pdf

Behavioural & social sciences study design

All studies must disclose on these points even when the disclosure is negative.

Study description	Our study includes experimental quantitative data. In this work, two groups of participants were asked to learn novel verb-object pairings at the beginning of a first session. They then immediately practiced those associations twice in each of three cycles, six times overall. Subjects in the retrieval group (n = 49) practiced by actively recalling the object image from memory when cued with the verb. Critically, in each of the three cycles they were asked to answer one conceptual and one perceptual question about the recalled object as fast as possible. Subjects in the restudy group (n = 24) instead practiced by re-encoding the intact verb-object pairings, and were similarly asked to answer a conceptual and a perceptual question about the object in each cycle, but they did so while seeing the object on the screen. All participants returned to the lab 48h later for a delayed cued recall test, where each verb-object pairing was probed once more with a conceptual and once with a perceptual question.
Research sample	Fifty-seven healthy volunteers from the local student population in Birmingham participated in the retrieval condition (45 female and 12 male, mean age [M] = 19.95, standard deviation [SD] =.79). Another 26 volunteers participated in the restudy group (21 female and 5 male, M =18.92, SD =.89). In our study, we intended to represent healthy, young adults which is presumed to be provided by our sample.
Sampling strategy	Previously published work has found an effect size of d=.55 for the perceptual-conceptual gap in RTs during retrieval (Linde-Domingo et al., 2019). We expected an effect size at least as large on day 2 in the repeated retrieval group. A power analysis in G*Power (Faul, Erdfelder, Buchner, & Lang, 2009) with d=.55, α= .05 and a power of 0.9 suggested that a sample size of at least 30 was required to detect an existing effect in the retrieval group. The effect of most interest in the retrieval group was a significant interaction between testing day and question type, specifically such that the gap between conceptual and perceptual RTs would significantly increase from day 1 to day 2. The power for this interaction contrast could not be estimated a priori from the work of Linde-Domingo et al. (2019). To have sufficient power to detect an increase in the conceptual-perceptual gap, we decided to double their sample size, aiming for 48 subjects in the retrieval group (see results section for corresponding posthoc power analyses).<br>The second comparison of interest in this study was a contrast between the conceptual-perceptual gap on day 2 (i.e., delayed test) in the retrieval and the restudy groups. Again, since the effect size could not be estimated directly from previous work, we aimed for n=24 participants in the restudy group based on (Linde-Domingo et al., 2019) using n=24 within multiple groups to do between group comparisons. We thus aimed for a sample size of n=72 overall for the critical comparison of the retrieval and the restudy group. Again, posthoc power analyses can be found in the results section.<br>Association pairings within the tasks were semi-randomized for each subject.
Data collection	A computer task was performed by subjects, and pen-and-paper responses were collected at the end of the experiment. Participants performed the task while a researcher was present in the same room. The researcher was not blind to the experimental group condition.
Timing	The data collection of the first group started in 2018 and ran until 2019, the data collection of the second group started in 2019 and ran until 2020.
Data exclusions	Eight subjects were excluded due to absence on the second testing day or missing data in the retrieval group. Two were excluded due to absence on the second testing day in the restudy group.
Non-participation	No participants declined participation. Absence on the second testing day was explained by a change of plans. In total, 10 subjects were excluded.
Randomization	We initially conducted the study for the first (repeated retrieval) group, and only started data collection for the second (restudy) group after finishing the data collection of the first group. This is because we needed response timing information from the first group to determine the trial timings in the second group, to keep the paradigm as similar as possible for both groups. Participants were assigned by choosing present volunteers at the specific data collection time period, and as such, no active randomization was performed on the assignment of participants to groups. However, under the only restriction of time difference between the groups, the recruitment within groups was performed randomly. Moreover, apart from the time difference, no confounding variables were obvious, as the testing between groups also happened over the same seasons.

Reporting for specific materials, systems and methods

We require information from authors about some types of materials, experimental systems and methods used in many studies. Here, indicate whether each material, system or method listed is relevant to your study. If you are not sure if a list item applies to your research, read the appropriate section before selecting a response.

Materials & experimental systems

n/a	Involved in the study	
☒	☐	Antibodies
☒	☐	Eukaryotic cell lines
☒	☐	Palaeontology and archaeology
☒	☐	Animals and other organisms
☐	☒	Human research participants
☒	☐	Clinical data
☒	☐	Dual use research of concern

Methods

n/a	Involved in the study	
☒	☐	ChIP-seq
☒	☐	Flow cytometry
☒	☐	MRI-based neuroimaging

Human research participants

Policy information about studies involving human research participants

Population characteristics	See above.
Recruitment	Healthy volunteers from the local student population in Birmingham signed up on the study participation system SONA and were selected randomly and anonymously. Except for the fact that we predefined the student population as our sampling source, within this population, no self-selection bias is obvious.
Ethics oversight	The research was approved by the STEM ethics committee of the University of Birmingham.

Note that full information on the approval of the study protocol must also be provided in the manuscript.
